# Editorial: Microbial resilience in plant nutrient management towards sustainable farming

**DOI:** 10.3389/fmicb.2023.1280811

**Published:** 2023-10-18

**Authors:** Manoj Kumar Solanki, Krishan K. Verma, Khondoker M. G. Dastogeer, Freddy Mora-Poblete, Sunil Mundra

**Affiliations:** ^1^Department of Life Sciences and Biological Sciences, IES University, Bhopal, Madhya Pradesh, India; ^2^Plant Cytogenetics and Molecular Biology Group, Faculty of Natural Sciences, Institute of Biology, Biotechnology and Environmental Protection, University of Silesia in Katowice, Katowice, Poland; ^3^Key Laboratory of Sugarcane Biotechnology and Genetic Improvement (Guangxi), Guangxi Key Laboratory of Sugarcane Genetic Improvement, Ministry of Agriculture, Sugarcane Research Institute, Guangxi Academy of Agricultural Sciences, Nanning, Guangxi, China; ^4^Department of Plant Pathology, Bangladesh Agricultural University, Mymensingh, Bangladesh; ^5^Institute of Biological Sciences, University of Talca, Talca, Chile; ^6^Department of Biology, College of Science, United Arab Emirates University, Al-Ain, Abu-Dhabi, United Arab Emirates; ^7^Khalifa Center for Genetic Engineering and Biotechnology, United Arab Emirates University, Al Ain, United Arab Emirates

**Keywords:** phosphite, biostimulant, continuous cropping, microbial dynamics, legume cultivation, optimized fertilizer strategies, sustainable farming, nutrient cycling and balance

Agriculture plays a pivotal role in the realm of global sustainability and food security. Recent scientific endeavors are concentrated on harnessing the latent potential of diverse microbial populations and their interactions to improve agricultural practices, enhance crop yields, and propel sustainable development (Pang et al., 2022; Solanki et al., 2023; Wang et al., 2023). Amidst the pursuit of sustainable agricultural practices, an intricate alliance between plants and their microbial counterparts has emerged as a cornerstone of innovation. With the global population continuing its rapid expansion, the imperative to ensure both food security and environmental equilibrium has become more acute than ever before. “*Microbial resilience in plant nutrient management towards sustainable farming*” delves profoundly into a pivotal domain of this agricultural revolution—the resilient interplay between microorganisms and plant nutrient management.

This editorial elucidates the urgency and significance of comprehending microbial resilience in the context of sustainable farming. It traverses a compendium of studies shedding light on innovative approaches within the realm of agricultural microbiology. These span from the application of novel fertilizers to the manipulation of microbial communities, all in pursuit of ameliorating soil health and elevating crop productivity. The delicate equilibrium between soil microorganisms and plant health profoundly influences nutrient uptake, disease resistance, and the overall vitality of the ecosystem. Advanced research delves into the dynamic mechanisms underlying microbial contributions to nutrient cycling, bioavailability, and the alleviation of plant stress. By unraveling the intricate interactions of microbial communities and their responses to changing agricultural practices, this compilation strives to chart a course of well-informed decisions that foster resilient strategies for nutrient management. Moreover, the editorial aims to fortify agricultural sustainability during an epoch characterized by escalating challenges.

## Phosphite: a potential fertilizer and biostimulant

Phosphorus is a vital nutrient for plant growth, and its efficient utilization in agriculture is crucial for optimal crop production. Phosphite, a soluble form of phosphorus, has emerged as a promising candidate for enhancing fertilizer efficiency and promoting plant health. Li et al. investigated the effects of phosphate and phosphite fertilizers on soil properties and microorganisms in alfalfa fields. Their study revealed that both fertilizers increased the total and available phosphorus content in the soil. Interestingly, soil treated with phosphite exhibited lower phosphorus levels than phosphate-treated soil, suggesting a potential for more controlled nutrient management. The study also unveiled a complex interplay between alkaline phosphatase (ALP) activity, bacterial communities, organic phosphorus, and pH. This research underscores the potential of phosphite as a sustainable agricultural tool with implications for enhanced crop yield and soil health.

## Continuous cropping and microbial dynamics

Continuous cropping is known to pose challenges to soil health and crop yield due to the depletion of nutrients and changes in microbial communities. Wang J. et al. investigated the impact of continuous cropping on melon rhizospheric microbial communities. Using high-throughput sequencing, they analyzed greenhouse rhizosphere soil from Jiashi muskmelon replanted over 0–3 years. The study revealed a dynamic microbial response to continuous cropping, with increased bacterial abundances but reduced richness and diversity over time. Interestingly, bacterial composition became more uniform after 2–3 years of continuous cropping, potentially indicating a shift toward more adapted communities. The research emphasized the need for informed soil management and fertilization strategies to mitigate the adverse effects of continuous cropping on microbial diversity and overall crop health.

## Addressing challenges in legume cultivation

Legume crops hold great promise in promoting sustainable agriculture and addressing global food security concerns. However, pathogens like *Rhizoctonia solani* pose a significant threat to legume production. A review by Akber et al. highlighted the challenges posed by *R. solani* in legume crops and explored advanced molecular techniques for accurate disease detection and management. The study underscored the importance of innovative detection methods such as PCR variants, magnetic-capture hybridization PCR, and loop-mediated isothermal amplification in overcoming the limitations of traditional approaches. This comprehensive review provides valuable insights into the distribution, infection mechanisms, and potential management strategies for *R. solani*, paving the way for improved legume cultivation practices.

## Cold-adapted microorganisms: a solution for straw degradation

Agricultural waste management is a critical aspect of sustainable agriculture. He et al. addressed the challenge of corn straw degradation by harnessing the capabilities of cold-adapted microorganisms. These organisms thrive at low temperatures and efficiently break down lignocellulosic materials. The study isolated high-efficiency cellulose-degrading bacteria and demonstrated their potential to enhance cellulose degradation efficiency. The results showcased the effectiveness of *Bacillus subtilis* K1 in promoting lignocellulose degradation and the humic acid-to-fulvic acid ratio during corn straw composting. This innovative approach presents a promising sustainable agricultural waste management solution to the persistent issue of crop residue utilization.

## Optimized fertilizer strategies for soil health

Excessive fertilizer use has been linked to soil degradation and environmental pollution. Wang M. et al. explored optimized fertilizer strategies to mitigate these challenges and enhance soil health. By evaluating various fertilizer combinations, including cow dung manure, chemical fertilizer, and their combinations, the study demonstrated significant effects on soil properties, cucumber nutrients, and microbial communities. Notably, eco-friendly approaches like reduced chemical fertilizer with manure improved bacterial abundance, fungal diversity, and nutrient availability. These findings highlight the potential of environmentally conscious fertilizer strategies in promoting carbon neutrality and sustainable agricultural practices.

## Enhancing nitrogen uptake in sugarcane

Sugarcane cultivation is essential for sugar and energy production, but the excessive use of synthetic nitrogen fertilizers raises environmental concerns. Guo et al. introduced *Enterobacter roggenkampii* ED5, an endophytic N-fixing bacterium, as a solution to enhance nitrogen uptake in sugarcane. Their study revealed that ED5 positively influenced nitrogen metabolism, enzyme activities, and microbial communities, particularly under varying nitrogen levels. The findings suggest that ED5 has the potential to support sustainable sugarcane production while reducing the overreliance on synthetic nitrogen fertilizers, thus contributing to agricultural sustainability.

## Biopriming for enhanced seed germination and stress resilience

Seed priming methods have gained attention for enhancing germination, vigor, and stress tolerance. Fiodor et al. focused on biopriming, a technique that utilizes beneficial microorganisms like plant growth-promoting bacteria (PGPB) to promote seed germination and manage biotic stress. The study screened diverse bacterial strains for their growth-promoting activities and revealed their potential to enhance germination, possibly through the solubilization of essential nutrients. This approach presents a sustainable alternative to chemical fungicides and underscores the role of microbial interactions in supporting plant growth and resilience.

## Mitigating acidification for sustainable sugarcane cropping

Sugarcane ratooning, a common practice for successive harvests, can lead to soil acidification and degradation over time. Khan et al. used a multidisciplinary approach in their recent study to investigate the effects of repeated sugarcane ratooning cycles on both soil characteristics and microbial communities. Notably, their research illuminated the complex interactions between soil characteristics, bacterial phyla, and microbial species. Importantly, the study emphasized the necessity of mitigating acidification through practices such as liming or biofertilizer application to ensure the long-term sustainability of sugarcane cropping and maintain soil health.

## Conclusion

In the pursuit of sustainable agriculture, advancements in agricultural microbiology offer valuable insights and solutions. The studies discussed in this summary collectively underscore the potential of innovative approaches, from novel fertilizers and microbial community management to seed priming techniques and waste utilization. By harnessing the power of microorganisms and their interactions, researchers contribute to the development of environmentally conscious and productive agricultural practices that support food security and long-term sustainability. As agriculture continues to evolve, these findings provide a solid foundation for addressing pressing challenges and advancing agricultural systems worldwide.

## Author contributions

MS: Conceptualization, Writing—original draft. KV: Writing—review and editing. KD: Writing—review and editing. FM-P: Writing—review and editing. SM: Writing—review and editing.
